# Flower-like Composite Material Delivery of Co-Packaged Lenvatinib and Bufalin Prevents the Migration and Invasion of Cholangiocarcinoma

**DOI:** 10.3390/nano12122048

**Published:** 2022-06-15

**Authors:** Zhouyu Ning, Yingke Zhao, Xia Yan, Yongqiang Hua, Zhiqiang Meng

**Affiliations:** Department of Integrative Oncology, Fudan University Shanghai Cancer Center, Shanghai 200032, China; yuzhou3065@126.com (Z.N.); ykzhao@fudan.edu.cn (Y.Z.); dryanxia@163.com (X.Y.); keqiang1215@126.com (Y.H.)

**Keywords:** Cholangiocarcinoma (CCA), lenvatinib, bufalin, nanoparticle, integrated traditional chinese and western medicine

## Abstract

The co-delivery of multiple drugs using nanocarriers has been recognized as a promising strategy for cancer treatment to enhance therapeutic efficacy. In this study, a monodisperse mesoporous silica nanoparticle (mSiO_2_) is prepared and functionalized into high-efficiency loaded Lenvatinib and Bufalin for targeted delivery to Cholangiocarcinoma (CCA). mSiO_2_ was synthesized on solid silica nanoparticles by oil–water interface method, and highly monodisperse mSiO_2_ with uniform morphology was obtained. mSiO_2_ was then sequentially modified by polyethylene glycol (PEG) and the targeting molecule folic acid (FA). mSiO_2_-FA was designed as co-delivery system for Lenvatinib (Le) and Bufalin (Bu) to increase drug availability and highly target tumor cells. Compared with unfunctionalized mSiO_2_, mSiO_2_-FA can more efficiently enter human CCA cell lines (9810 cells) and enhance intracellular drug delivery. Moreover, drug-loaded mSiO_2_-FA (Le/Bu@mSiO_2_-FA) significantly inhibited the viability, migration and invasion of 9810 cells. In vivo, the nanocomplex significantly reduced the tumor load in CCA tumor-bearing mouse models compared to Le or Bu alone. The current work provides a useful strategy for highly targeted and multidrug-resistance reversal therapy for CCA.

## 1. Introduction

Morbidity and mortality of cholangiocarcinoma (CCA) have increased year by year [1]. Most patients are in advanced stage when they first visit the doctor, and the recurrence rate after CCA is high. Even after radical resection, the 5-year survival rate for CCA patients ranges from 22% to 44% [2]. Many studies have used gemcitabine combined with cisplatin and gemcitabine combined with albumin-bound paclitaxel as the first-line standard regimens for advanced or metastatic CCA [3,4]. CCA-targeted therapies targeting EGFR, RAS/RAF/MEK/MAPK, PI3K/Akt/mTOR signaling pathways, anti-angiogenic therapeutic targets, IDH_1/2_ mutations, HER_2_ amplification, FGFR_2_ fusion and NTRK fusion have made significant progress, but most of them are currently in the stage of research and development and clinical studies [5,6]. Targeted PD-1 therapy has been approved for advanced intrahepatic cholangiocarcinoma (ICC) in cases where there is a mismatch repair deficiency (d MMR) or high micro satellite instability (MSI-H). The efficacy of immune-checkpoint therapy in p MMR/MSS type ICC is not clear, and the predictive effect of TMB and PDL-1 expression state on the efficacy is not yet confirmed [7,8]. At present, CCA immunotherapy-related studies are mostly in the pre-clinical and clinical trial stage, and significant clinical benefits have not been obtained, which may be related to the heterogeneity and unique tumor microenvironment of CCA tumor rejection antigen (TRA). Therefore, finding novel strategies for ICC therapy is urgent.

Clinical studies confirmed that proper combinations of Chinese medicine (CM) and Western medicine (WM) could decrease toxicity and increase efficiency for promoting strengths and avoiding weaknesses [9,10,11]. Integrated Traditional and Western Medicine has shown some advantages in the treatment of CCA, especially in middle and advanced stage CCA [12,13]. Lenvatinib is a tyrosine kinase receptor (RTK) inhibitor that inhibits kinases including vascular endothelial growth factor receptor VEG-FR1 (FLT1), VEGFR2 (KDR), and VEGFR3 (FLT4) [14,15]. It also inhibits other RTKs associated with pathogenic tumor angiogenesis, tumor cell growth, and tumor development for normal cellular functions, including fibroblast growth factor receptors (FGFR) 1, 2, 3 and 4, KIT, platelet-derived growth factor receptor-α/β (PDGFR-α/β) and RET [16,17]. For advanced hepatic carcinoma (HCC) phase II trial results showed that Lenvatinib was superior in terms of overall survival, as well as significantly improved progression-free survival (PFS), time to tumor progression (TTP) and objective response rate (ORR), which is the first-line treatment for HCC [18]. However, CCA is highly desmoplastic and its tumor microenvironment is exceptionally rich, features that determine its resistance to the chemotherapeutic agent Lenvatinib, limiting the therapeutic efficacy of Lenvatinib [19,20]. In addition, the main bioactive component extracted from toad venom, Bufalin, has been proven to be an effective antitumor drug [21,22,23]. Bufalin effectively kills tumor cells by three mechanisms: inhibiting cell proliferation, promoting apoptosis, reversing tumor cell multidrug resistance, and inhibiting tumor vessel proliferation [24,25,26].

In recent years, multifunctional nanocomposites with simultaneous diagnostic, targeting and therapeutic functions have drawn much attention in cancer therapy and tumor suppression [27,28]. Nanomaterials have developed rapidly and nanotechnology has been applied in medical fields such as cancer and cardiovascular medicine [29]. The targeted drug delivery system has drawn more and more attention in cancer therapy over the past few decades. For a novel drug delivery system, drug design and development are crucial aspects [30]. Compared with the traditional single-drug treatment, the use of nanomaterials as carriers through the use of mixed drugs can greatly improve the efficacy of the drug [31,32]. Because of its unique and excellent properties in drug-delivery system, virus-like mesoporous silica nanoparticle has become a hot topic in the medical field nowadays [19,33].

**Figure 1:**
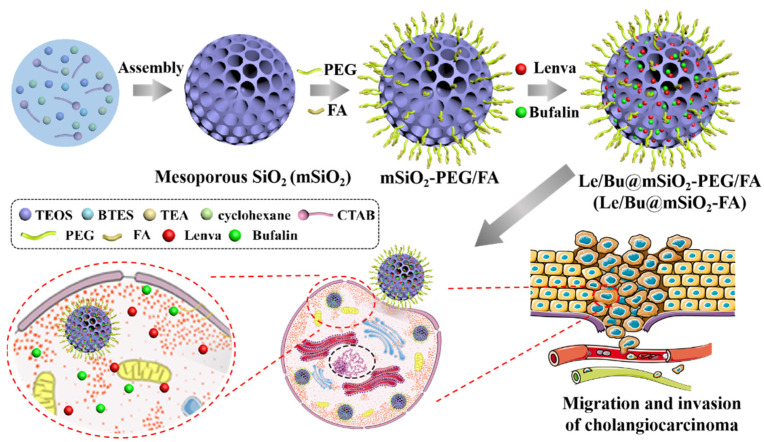
The synthesis process of Le/Bu@mSiO_2_-FA.

Here, we successfully developed a controlled release nanocarrier system of mesoporous silica nanoparticles (mSiO_2_-FA) for co-delivery of Le and Bu (Figure 1). mSiO_2_ was synthesized on solid silica nanoparticles by oil–water interface method, and highly monodisperse mSiO_2_ with uniform morphology was obtained. mSiO_2_ was then sequentially modified by polyethylene glycol (PEG) and the targeting molecule folic acid (FA) to study the loading rate and release rate of the co-loaded Le and Bu. In vitro drug release experiments were conducted to study the release regularity of Le and Bu. In addition, the biological evaluation of human CCA cell lines (9810 cells) was carried out by mSiO_2_-FA analysis of cell uptake, cell viability, migration and invasion. Finally, we evaluated the in vivo anti-tumor effect of mSiO_2_-FA in the tumor-bearing mouse model.

## 2. Materials and Methods

### 2.1. Materials

Tetraethyl orthosilicate (TEOS), ammonia water, ethanol, hexadecyltrimethylammonium bromide (CTAB), Triethanolamine (TEA), cyclohexane solution, ammonium nitrate (NH_4_NO_3_), N-Hydroxysuccinimide (NHS), 3-(ethyliminomethylideneamino)-N,N-dimethylpropan-1-amine,hydrochloride (EDC), PEG-COOH and FA, were purchased from Aladdin (Shanghai, China), Bufalin (Bu) and Lenvatinib (Le) were purchased from GLPBIO (Montclair, CA, USA), Fluorescein Isothiocyanate (FITC), 4′,6-diamidino-2-phenylindole (DAPI), and hematoxylin and eosin were acquired from Solarbio (Beijing, China), 9810 cells were detected by Cell Bank of Chinese Academy of Sciences (Shanghai, China), fluorescent sealing solution were purchased from Merck Millipore (Billerica, MA, USA). The Cell Counting Kit-8 (CCK-8) assay kit were purchased from Beyotime (Shanghai, China). Transwell chambers and Matrigel were obtained from Corning (Corning, NY, USA). Shanghai Model Organisms Center, Inc (Shanghai, China) provided us the BALB/c nude mice. Hematoxylin and eosin (H&E) were obtained from Sangon (Shanghai, China). Alanine aminotransferase (ALT) and aspartate aminotransferase (AST) ELISA kits were purchased from mlbio (Shanghai, China).

### 2.2. Preparation of Mesoporous Silica (mSiO_2_) Nanoparticles

Mesoporous silica nanoparticles (mSiO_2_) were synthesized by oil–water interface method. Briefly, 6 g CTAB and 0.18 g TEA were added to a single-mouth round-bottomed flask (100 mL) containing 60 mL water and stirred gently at 60 °C for 1 h. Then, 20 mL of cyclohexane solution with 20 *v/v* % TEOS was dropped onto the upper layer of CTAB aqueous solution and stirred at 60 °C for 12 h. After the reaction, the aqueous phase was extracted and centrifugated at a high speed (15,000 Revolutions Per Minute (rpm), 30 min), the supernatant was discarded, the products were added to 50 mL 0.6 wt. % ammonium nitrate (NH_4_NO_3_) ethanol solution, and the surfactant CTAB was extracted by reflux at 60 °C, and the extraction was repeated 3 times, for 12 h each time.

### 2.3. Modification of mSiO_2_

Ultrasonic dispersion of 20 mg amino mSiO_2_ into 20 mL ethanol was conducted. Then, 20 mg NHS, 15 mg EDC, and 10 mg PEG-COOH were added and stirred for 24 h, and mSiO_2_-PEG was obtained by centrifugation. mSiO_2_-PEG was again dispersed into 20 mL ethanol, and 10 mg NHS, 15 mg EDC, and 2.0 mg FA were added and stirred for 24 h. The obtained products were separated by centrifugation, washed with ethanol, and then dried in vacuum for subsequent research.

### 2.4. Drug Loading and Releasing

Bufalin (Bu) and Lenvatinib (Le) were simply loaded into mSiO_2_-FA. In short, 10 mg mSiO_2_-FA nanoparticles were dissolved with PBS (20 mL, pH 7.4) by the ultrasonic bath, but Bu, Le, or Bu and Le were diffused with 2 mL water. Then, the solution containing the drug was mixed with mSiO_2_-FA nanoparticle solution, and stirred for the next 24 h. Next, drug-loaded nanoparticles were acquired through ultracentrifugation at (12,000 rpm) and washed by H_2_O 3 times, 5 mL each time, to detach free and surface-attached drugs. To measure the drug content, the supernatant was collected totally. Meanwhile, the UV-vis absorption spectra of the supernatant collected after the first centrifugation procedure was measured to determine the loading capacity of Le and Bu on mSiO_2_-FA. Drug loading efficient (DLE) was calculated as follows: DLE% = (weight of drug loaded/weight of fed drug initially) × 100. Drug loading content (DLC) was calculated as follows: DLC% = weight of drug loaded/weight of whole nanoparticles × 100.

The release rates of Bu and Le containing PBS (pH =7.4) were studied by dialysis. mSiO_2_-FA carrying Bu and Le were dispersed in 2 mL, 0.1 mol/L PBS then put in the dialysis bag. The dialysis bag was immersed in the same PBS with 18 mL at 37 °C, and the released medium was mixed at 80 rpm for 32 h. Samples from multiple time intervals (0, 1, 2, 3, 4, 5, 8, 12, 36, and 72 h) were taken then replaced by the same amount of new PBS. The concentration of each drug in the sample was measured with UV-Vis spectroscopy.

### 2.5. Cell Culture and Transfection

Human CCA cell line (9810) was cultured in DMEM containing 10% FBS and 1% penicillin/streptomycin (Sigma, St. Louis, MO, USA). After 24 h, before the incubation in the 5% CO_2_ incubator at 37 °C, 1 mg/mL mSiO_2_-FA-FITC was added by 2 μL to each well. Meanwhile, mSiO_2_-FITC was also transfected as a control. After the 9810 cells were incubated for 1, 2, and 6 h respectively, they were fixed by 4% paraformaldehyde for 15 min. For staining, DAPI was added into each plate to co-incubate the cells. Subsequently, a drop of fluorescent sealing solution was added, and the cells were directly imaged with confocal laser scanning microscopy (CLSM, Leica, Wetzlar, Germany).

### 2.6. CCK-8 Assay

For detecting the effects of nanoparticles and concentrations on 9810 cell viability, we used the Cell Counting Kit-8 (CCK-8) assay kit. In brief, after seeding 9810 cells into 96-well plates (1 × 10^4^ per well), they were incubated for 24 h with series concentrations of mSiO_2_-FA (0, 10, 20, 40, 80, 160, and 200 μg/mL), Le, Bu, Le/Bu, or Le/Bu@mSiO_2_-FA. After that, we added 10 µL of the CCK-8 solution into the medium with cell culture and incubated it for another 1 h. The absorbance was assessed at a wavelength of 450 nm using a microplate reader (Bio-Tek, Norcross, GA, USA).

### 2.7. Wound-Healing Assay

We used wound healing assay to assess cells migrating. Briefly, the 9810 were seeded in 6-well plates (2.5 × 10^5^ per well). A sterile pipette tip was used to make a scratch after cells were attached to the well bottom, then the width of the original scratch was recorded. Fresh serum-free DMEM medium substituted the residual liquid in the wells, added with free mSiO_2_-FA nanoparticles, free Le, Le@mSiO_2_-FA, free Bu, Bu@mSiO_2_-FA, Le/Bu@mSiO_2_-FA (100 μg/mL), respectively. Cells were cultured at 37 °C for 24 h. The width of the scratch was then measured and recorded.

### 2.8. Apoptosis Analysis

Apoptosis analysis was performed using Annexin V-FITC Apoptosis Detection Kit (Beyotime, Shanghai) by the manufacturer’s instructions. In brief, 9810 cells were treated with free mSiO_2_-FA nanoparticles, free Le, Le@mSiO_2_-FA, free Bu, Bu@mSiO_2_-FA, free Le/Bu, Le/Bu@mSiO_2_-FA (100 μg/mL) for 48 h. Lastly, the cells were double stained with PI and Annexin V-FITC, and cell apoptosis was measured with flow cytometer (Thermo Fisher Scientific, Waltham, MA, USA).

### 2.9. Endocytosis Pathway Assay

To determine the endocytosis pathway of Le/Bu@mSiO_2_-FA nanoparticles, 9810 cells were inoculated on a 6-well plate at a density of 10^5^ cells per well for 2 days at 37 °C and cultured with various specific inhibitors for various endocytosis for 1 h at 37 °C. Afterwards, the uptake study was performed in the presence of the inhibitor and Le/Bu@mSiO_2_-FA at concentration of 50 μg/mL for 2 h at 37 °C. The supernatant was taken and washed with cold HEPES 3 times and analyzed using flow cytometry.

### 2.10. Transwell Assay

In transwell chambers with 8 μm pore size and Matrigel coating, the invasion of 9810 cells was evaluated. A total of 3 × 10^4^ 9810 cells were suspended in 100 μL serum-free DMEM medium, then added to the upper chamber of a transwell plate. Subsequently, free Le, free Bu, Le/Bu, or Le/Bu@mSiO_2_-FA (100 μg/mL), respectively, were added. Besides, the lower chamber was added 800 μL DMEM medium including 80 μL FBS. Before cells in the lower chamber were fixed by methanol and then 0.2% crystal violet-stained, they were incubated 24 h at 37 °C with 5% CO_2_. The invaded cells were counted by utilizing a light microscope (Zeiss, Jena, Germany). To assess the migration of 9810 cells, all the procedures were identical to invasion assay, while the chamber was not covered by Matrigel and the incubation time was extended to 36 h.

### 2.11. Animal Experiments

BALB/c nude mice (male; 4–6 weeks; 18–22 g) were selected from Shanghai Model Organisms Center, Inc. The Ethics Committee in Fudan University Shanghai Cancer Center certified the animal experiment. We inoculated 9810 tumor cells (1 × 10^6^) subcutaneously to the mice in their left armpit. When tumor volumes reached 40 mm^3^ in average, five groups were used to divide the tumor-bearing mice (*n* = 6 per group). In addition to the first group of the caudal vein injection of saline (as a control), the Le, Bu, Le/Bu, or Le/Bu@mSiO_2_-FA (10 mg/kg) were injected, respectively, into the other four group of mice. Every mouse was measured the body weight and all tumors were removed and photographed 21 days later. The long and short diameter sizes of tumors in nude mice were measured and recorded every day. Tumor volume size V = (tumor long diameter)/(tumor short diameter)^2^/2.

### 2.12. Histological Staining

The major organs (heart, spleen, kidney, lung, and liver) of the tumor-bearing mice in different groups of treating were collected, dehydrated, embedded, and stained with hematoxylin and eosin. We observed the histological changes under a light microscopy at 200× magnification (Olympus, Tokyo, Japan).

### 2.13. Measurement of Serum Biochemical Index in Blood

The blood serum biochemical index was used to evaluate the long-term accumulation of Le/Bu@mSiO_2_-FA nanoparticles and drugs. The blood was collected by extracting eyeballs from different mice. Blood samples were analyzed through the blood cell analyzer, then the serum alanine aminotransferase (ALT) and aspartate aminotransferase (AST) were further detected.

### 2.14. Statistical Analysis

Statistical analyses were carried out with the SPSS 17.0 software (IBM, Armonk, NY, USA). We repeated every experiment independently by three times. All values are presented in the way of mean ± standard deviation (SD). Among them, values from multiple groups were compared via one-way ANOVA, and two groups were compared via Student’s *t*-test. *p* < 0.05 indicated statistical significance.

## 3. Results and Discussions

### 3.1. mSiO_2_-FA Synthesis and Characterization

**Figure 2:**
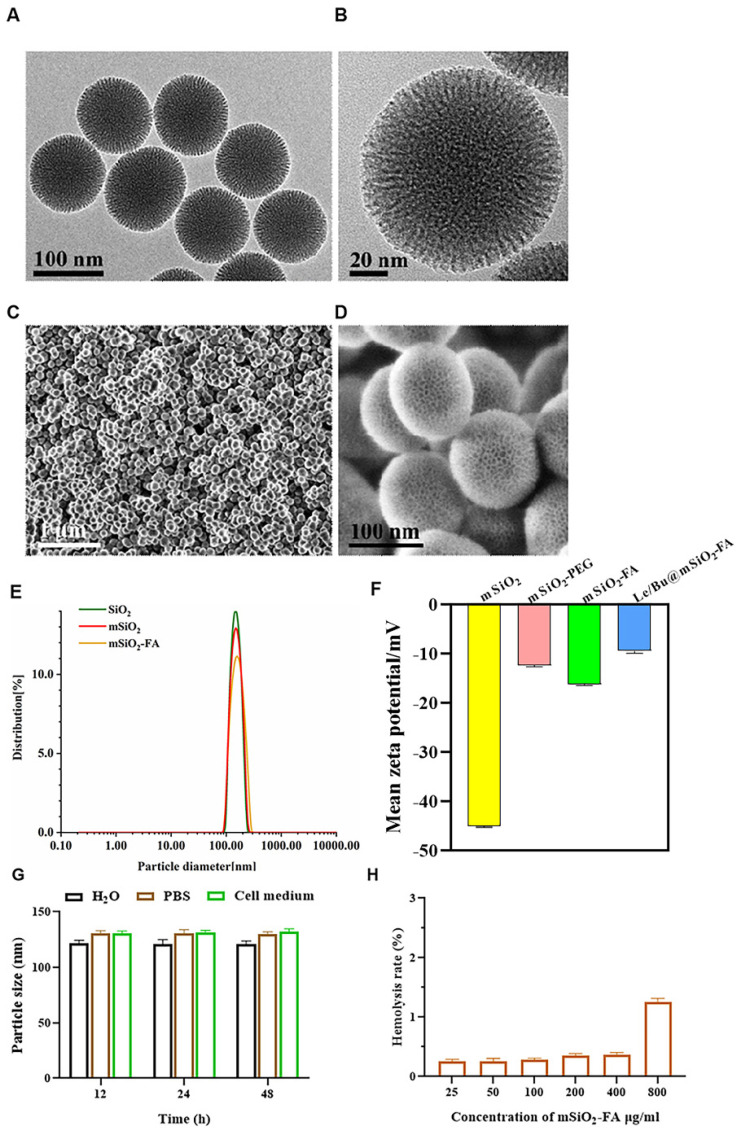
Characterization of mSiO_2_-FA. (**A**,**B**) TEM image of mSiO_2_-FA nanoparticles. Scale bars = 100 nm (**A**) and 20 nm (**B**) for TEM. (**C**,**D**) SEM image of mSiO_2_-FA nanoparticles. Scale bars = 1 μm and 100 nm for SEM. (**E**) Size distribution of the SiO_2_, mSiO_2_, and mSiO_2_-FA nanoparticles by Dynamic light scattering (DLS) assay. (**F**) Zeta potential of the mSiO_2_, mSiO_2_-FA, and Le/Bu@mSiO_2_-FA nanoparticles. (**G**) Size distributions of mSiO_2_-FA nanoparticles in different medium for different time. (**H**) Hemolysis test results of mSiO_2_-FA nanoparticles. TEM—Transmission Electron Microscope; SEM—Scanning Electronic Microscopy; Le/Bu—Lenvatinib and Bufalin combination.

The synthetic process of Le/Bu@mSiO_2_-FA was summarized in Figure 1. mSiO_2_ was synthesized on solid silica nanoparticles by oil–water interface method, and highly monodisperse mSiO_2_ with uniform morphology was obtained. mSiO_2_ was then sequentially modified by polyethylene glycol (PEG) and the targeting molecule folic acid (FA). The TEM and SEM images of the obtained mSiO_2_-FA show that the nanoparticles were monodisperse, spherical in shape with an average diameter of 115–120 nm (Figure 2A–D). The size distribution of Solid SiO_2_, mSiO_2_, and mSiO_2_-FA was detected by dynamic light scattering (DLS) assay, and the results showed that the particle size of the three groups did not change much, with a diameter of 120 ± 23 nm (Figure 2E). To detect the modification process, the charge of these nanoparticles on the surface was also measured (Figure 2F). The mSiO_2_ exhibited a strongly negative surface (−44.3 ± 0.2 mV). The charge of PEG-modified nanoparticles was −12.4 ± 0.42 mV. The final product mSiO_2_-FA had a negative surface too, the zeta potential of which was −10 ± 0.2 mV, showing that mSiO_2_-FA is physically stable, and the synthesis of Le/Bu@mSiO_2_-FA is successful. In order to determine the stability of mSiO_2_-FA nanoparticles under physiological conditions, the nanoparticles were placed in water (H_2_O), phosphate buffer (PBS) and culture medium for 12, 24, and 48 h, and then the size of nanoparticles was measured by dynamic light scattering. The results showed that the average size of mSiO_2_-FA nanoparticles stayed at about 120 nm; nevertheless, the incubation time was 48 h, illustrating that these nanoparticles were strongly stable (Figure 2G). In addition, the hemolysis ratio of mSiO_2_-FA with different concentrations was also tested in vitro (Figure 2H). The mSiO_2_-FA induced a hemolysis rate of less than 2.0% by contacting with erythrocytes in whole blood at 37 °C for 1 h (clinical requirements for biomedical materials are lower than 5.0% of hemolysis rate). Therefore, the mSiO_2_-FA was considered as a nonhemolytic material.

### 3.2. Drug Loading and Release of mSiO_2_-FA

**Figure 3:**
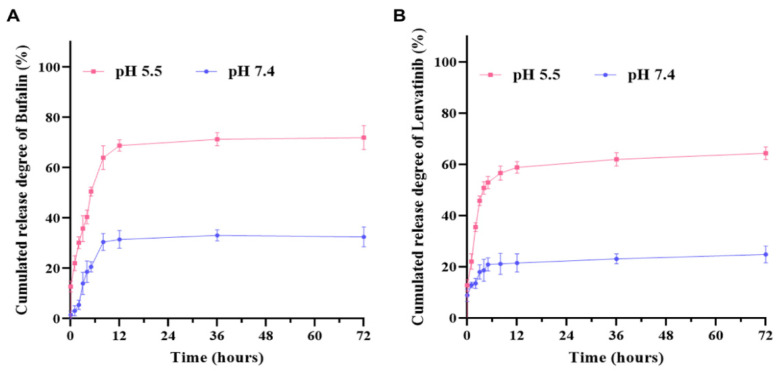
(**A**) Bu release profiles of Le/Bu@mSiO_2_-FA in different conditions; (**B**) Le release profiles of Le/Bu@mSiO_2_-FA in different conditions. Le, Lenvatinib; Bu, Bufalin; Le/Bu, Lenvatinib and Bufalin combination.

Because of the mesoporous structure and large specific surface (Table 1) area of mSiO_2_, we used mSiO_2_ to load bufalin and Lenvatinib. In this study of Len loading ability of solutions with distinct drug concentration, DLE (~95.3%) was still high even when the concentration of Le achieved 100 μg/mL, illustrating a high affinity in Le with mSiO_2_-FA nanoparticles. Moreover, when Le concentration was more than 500 μg/mL, a relatively high concentration, it could lead to a DLC of about 40.3%, which is also relatively high. The results (Table 2) indicated that the DLE of Le was not interfered with by the Bu addition when Le was loaded. With the concentration increasing, the Bu loading ability raised to 39.2%, however, the drug loading efficiency decreased slightly when the Bu concentration was changed to 500 μg/mL (80.3%, compared with 89.3% in 250 μg/mL and DLC of 17.4%). In addition, in further studies, we selected mSiO_2_-FA nanoparticles loaded Le and Bu with DLC of 16.4% (DLE of 90.1%) and 17.4% (DLE of 89.3%), separately. Controlled drug release in tumor tissue is one of the keys of drug delivery system for tumor therapy. Tumors are known to have a pH (about 6) slightly lower than normal tissue and blood. In addition, the pH of normal human plasma is about 7.35–7.45. Therefore, we performed pH-stimulated drug release of Le/Bu@mSiO_2_-FA in vitro. The percentage of Bu and Le released by Le/Bu@mSiO_2_-FA nanoparticles under different pH conditions is shown in Figure 3. At physiological pH (~7.4), mSiO_2_-FA released about 33.47% Bu after 72 h, while in tumor cell environment (pH 5.5), up to 74.27% Bu could escape from the nanoparticles, suggesting that the dismantling of nanoparticles can greatly promote the release of Bu (Figure 3A). The release behavior of Le was similar to that of Bu, but the release rate was relatively slow (Figure 3B). In clinical use, sustained-release drugs can stabilize the concentration of the drug in the body, thus achieving a lasting effect and avoiding the side effects associated with the concentration of the drug. FA can also lead to more solid tumor deposition by enhancing permeability and retention. This property not only improves the targeting property of nanomaterials, but also enhances their sustained-release effect.

### 3.3. Le/Bu@mSiO_2_-FA Weakens the Viability, Migration and Invasion of CCA In Vitro 

**Figure 4:**
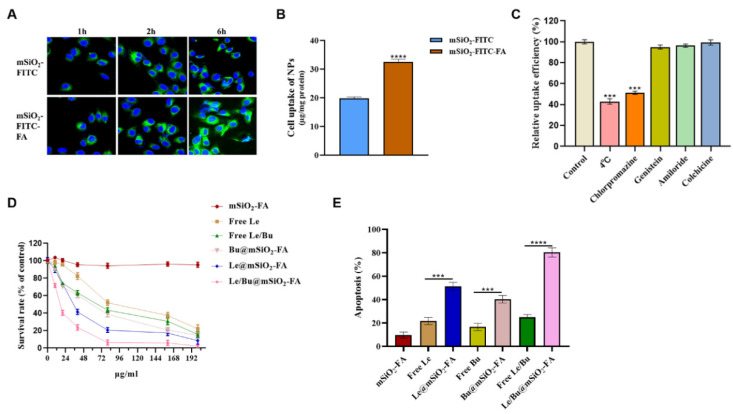
(**A**) Confocal laser scanning microscopy (CLSM) images of the 9810 tumor cells incubated with the same concentration (100 mg/mL) of mSiO_2_ and mSiO_2_-FA for 1, 2, and 6 h. Blue indicates DAPI nuclear stain, green is FITC fluorescence. (**B**) Uptake of mSiO_2_ and mSiO_2_-FA nanoparticles in 9810 cells at 6 h. (**C**) Relative uptake efficiency of mSiO_2_-FA nanoparticles in 9810 cells in the presence of various endocytosis inhibitors. (**D**) In vitro cytotoxicity of mSiO_2_-FA, free Le, free drugs Bu-Le combination, Bu@mSiO_2_-FA, Le@mSiO_2_-FA, Bu/Le@ mSiO_2_-FA by CCK8 assay. (**E**) Cell apoptosis of mSiO_2_-FA, free Le, Le@mSiO_2_-FA, free Bu, Bu@mSiO_2_-FA, free drugs Bu-Le combination, Bu/Le@ mSiO_2_-FA by flow cytometry. N = 3. *** *p* < 0.001, **** *p* < 0.0001. CLSM—confocal laser scanning microscopy; Le—Lenvatinib; Bu—Bufalin; Le/Bu—Lenvatinib and Bufalin combination.

Nanomaterials should be designed to enter cells rapidly and efficiently to realize a better therapeutic effect. Here, cell internalization of two types of FITC-labeled nanoparticles, mSiO_2_ and mSiO_2_-FA, were examined against 9810 cells by CLSM (Figure 4A). The relative uptake rate for each nanostructure was found to be quite different. After incubating for 1 h, the mSiO_2_ and mSiO_2_-FA nanoparticles obtained above were all able to penetrate the cell membrane of 9810 cells and accumulated throughout the cytosol. With the extension of time, the luminescence signal of mSiO_2_-FA nanoparticles entering 9810 cells was stronger than that of mSiO_2_ nanoparticles. It revealed that the internalization rate of mSiO_2_-FA nanoparticles is faster than that of mSiO_2_ nanoparticles. We further determined the relative uptake of mSiO_2_ and mSiO_2_-FA nanoparticles in 9810 cells after 6 h incubation (Figure 4B). The uptake of mSiO_2_-FA (32 μg/mg) in 9810 cells was significantly higher than that of mSiO_2_ (20 μg/mg). The results showed that, consistent with the results observed by the laser confocal microscope, the mSiO_2_-FA nanoparticles designed and synthesized by us could enter cells more efficiently and quickly, showing great application potential in the field of biomedicine. This is due to the use of FA here as a targeted agent in the drug delivery system. Folate receptor is a cell surface receptor that is overexpressed in various cancer cells. This receptor is closely related to folic acid (FA), the soluble form of the vitamin B [34]. FA can interact with folate receptors, resulting in cell internalization of anticancer drug loaded nanomaterials [35]. At present, the research on FA modified mesoporous silica surface as tumor cell targeting agent has been relatively mature [36,37,38,39,40,41,42,43].

In order to determine the endocytosis mechanism of mSiO_2_-FA nanoparticles composite by 9810 cells, the flow cytometry was performed (Figure 4C). Hypothermia or chlorpromazine, an inhibitor of endocytosis mediated by clathrin, obviously inhibited cellular uptake compared with the control experiment without inhibitor. While cells were pretreated by genistein (an inhibitor of endocytosis relied on caveolae), colchicine (an inhibitor of macropinocytosis relied on microtubule) and amiloride (an inhibitor of macropinocytosis), little or almost no inhibition was induced. This result showed that the cellular uptake pathway for vesicles was an endocytosis process that depended on energy and was mediated by clathrin. In 9810 cells, CCK-8 assay detected the cytotoxicity of various pharmaceutical preparations and vectors of increasing concentrations (0~200 µg/mL) for 48 h. With the increase of dose, all the cytotoxicity of pharmaceutical preparations to 9810 cells was enhanced (Figure 4D). The vector, mSiO_2_-FA, was nontoxic at a relatively high dose (200 µg/mL), proving that its biocompatibility was good. The killing potency of the free Lenvatinib or Bufalin to 9810 cells was very weak with low doses, as expected. Meanwhile, Lenvatinib@mSiO_2_-FA and Bufalin/Lenvatinib@mSiO_2_-FA were much more efficient compared with relevant groups (free Lenvatinib and free Bufalin/Lenvatinib combination) since cells could internalize the mSiO_2_-FA nanoparticles by clathrin-mediated energy-dependent endocytosis. Table 3 was the summary of the IC50 values (half maximal inhibitory concentration) which were calculated through the dose–response curve. The results indicated that the IC50 values of Bufalin/Lenvatinib@mSiO_2_-FA were much lower than free Lenvatinib, Lenvatinib@mSiO_2_-FA, Bufalin@mSiO_2_-FA and free drugs Bufalin/Lenvatinib combination, illustrating that Bufalin/Lenvatinib@mSiO_2_-FA had more efficiency for killing 9810 cells. The IC50 value of free Lenvatinib was 43.26 µg/mL. When the mSiO_2_-FA nanoparticles loaded Lenvatinib, the IC50 values reduced significantly, only around half of free Lenvatinib. A combination index (CI) is generally applied for analysis of the additive, synergistic or antagonistic effects of two drugs [44]. The CI could be obtained from the following formula: CI = C1/Cx1 + C2/Cx2, where C1 is the concentration of drug 1 required to induce a specific effect for combination therapy, Cx1 is the concentration of drug 1 generating the same influence independently; C2 is the concentration of drug 2 needed to reach the specific effect in combination therapy, Cx2 was the concentration of drug 2 achieving the identical influence individually. If CI > 1, it represents an antagonistic effect; if CI = 1, the two drugs show an additive effect; and if CI < 1, they have synergistic effects. Within our investigation, CI was calculated by IC50 values from Lenvatinib@mSiO_2_-FA, Bufalin@mSiO_2_-FA and Bufalin/Lenvatinib@mSiO_2_-FA. In 9810 cells, IC50 from Lenvatinib@mSiO_2_-FA and Bufalin@mSiO_2_-FA was 21.52 µg/mL and 22.17 µg/mL, separately (Table 3). For their combination therapy, Lenvatinib and Bufalin both had a concentration of 7.62 µg/mL, when the survival rate reached 50%. Consequently, nanogels delivered the Lenvatinib and Bufalin with the CI of 7.62/21.52 + 7.62/22.17 = 0.70 implying a synergistic effect. Cell apoptosis analysis was used to further verify the anti-cancer activity from our drug-loaded nanoparticles. In this study, the cells with Lenvatinib@mSiO_2_-FA and Bufalin/Lenvatinib@mSiO_2_-FA treatment had apoptosis rates of 51.28% and 80.26%, separately, while only 21.61% of cells were apoptotic after being treated with the same concentration of free Lenvatinib but the empty nanoparticles treated the cells with almost no apoptosis, which implies their biocompatibility (Figure 4E). These results confirmed that Bufalin/Lenvatinib@mSiO_2_-FA was more efficient at cytotoxicity than that of free Lenvatinib against 9810 cells.

**Figure 5:**
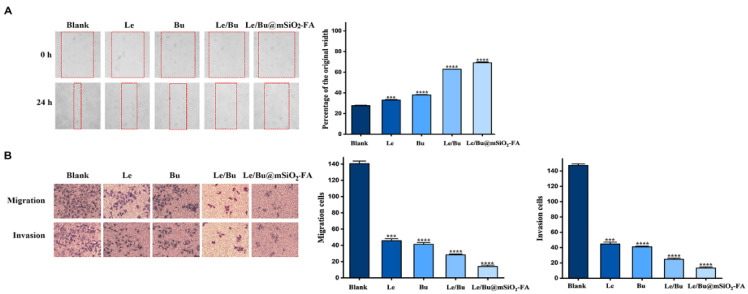
(**A**) Scratch assay of 9810 cells (control, free Le treatment, free Bu treatment, Le/Bu combination treatment, and Le/Bu@mSiO_2_-FA treatment) at 0 and 24 h (N = 3). (**B**) The cell migration and invasion of 9810 cells treated with control, free Le, free Bu, Le/Bu combination, and Le/Bu@mSiO_2_-FA for 24 h was detected by Transwell assay (N = 3). The invading and migrating cell numbers were then quantified by counting and depicted as bar charts. *** *p* < 0.001, **** *p* < 0.0001. Le—Lenvatinib; Bu—Bufalin; Le/Bu—Lenvatinib and Bufalin combination.

To evaluate the effects of Le/Bu@mSiO_2_-FA nanoparticles on the collective migration of CCA cells, we performed a 2D scratch assay on the monolayers of 9810 cells. After the introduction of a “scratch” or “wound” into the cell culture, cancer cells collectively migrated into the empty space, and images were taken immediately and 24 h after scratching for 9810 in Figure 5A. The results showed that 9810 cells in the control group exhibited significantly different wound-healing abilities compared to those treated with either Le or Bu alone, while the Le and Bu combination treatment further reduced the wound-healing ability of cancer cells. Moreover, Le/Bu@mSiO_2_-FA nanoparticles treatment resulted in the lowest wound-healing ability of cancer cells. In order to examine the ability to migrate and invade for 9810 cells with treatments of the single Le or Bu, the Le and Bu combination, or Le/Bu@mSiO_2_-FA nanoparticles, the Transwell assay was performed. Both Le and Bu-exposed cells demonstrated a significant decrease in cell migration and invasion as compared to control group. Likewise, Le and Bu combination-exposed cells exhibited a decrease in cell migration and invasion as compared to the single Le or Bu-treated group, but to a higher extent than the Le/Bu@mSiO_2_-FA nanoparticles-exposed cells (Figure 5B). These results indicate that Le/Bu@mSiO_2_-FA nanoparticles can attenuate the migration and invasion of CCA cells.

### 3.4. Le/Bu@mSiO_2_-FA Inhibits the Tumor Growth of CCA In Vivo

**Figure 6:**
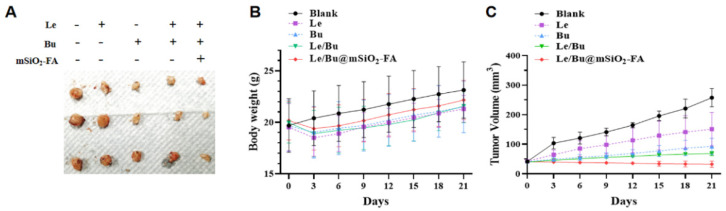
(**A**) Photographs of excreted tumor after different treatments. (**B**) Body weight change of treated mice of different treatments during treatment periods. (**C**) Tumor volume statistics of each group of tumor-bearing mice. N = 3 of each group.

Based on the above results, Le/Bu@mSiO_2_-FA could alleviate CCA migrating and invading in vitro, and the influence of Le/Bu@mSiO_2_-FA on CCA tumor growth in vivo was further explored. Orthotopic xenograft nude mice model of CCA was established by injecting 1 × 10^6^ 9810 cells into liver. The nude mice were injected into the caudal vein with single Le or Bu, Le/Bu, or Le/Bu@mSiO_2_-FA nanoparticles (100 μg/mL). Experimental mice show no significant change in body weight when they were treated with the drugs (Figure 6B), indicating that the treatments could be tolerated well and not lead to undesired effects on mice. Correspondingly, we detected the tumor size during the following 3 weeks after the treatments. Single Le or Bu slightly inhibits tumor growth of CCA, while the combination of Le and Bu further inhibits tumor growth. In contrast, Le/Bu@mSiO_2_-FA nanoparticles show significant anti-cancer effect (Figure 6A). Accordingly, tumor volume was monitored for 3 weeks after treatment. Le and Bu alone have little effect on tumor growth, which may be related to the poor retention effect of free Le and Bu on tumor. However, Le/Bu@mSiO_2_-FA group showed significant inhibitory effect (Figure 6C). To sum up, the results illustrate that Le/Bu@mSiO_2_-FA nanoparticles could inhibit the growth of tumors with high efficiency through their high DLC and targeted ability.

### 3.5. mSiO_2_-FA Has Good Biological Safety

**Figure 7:**
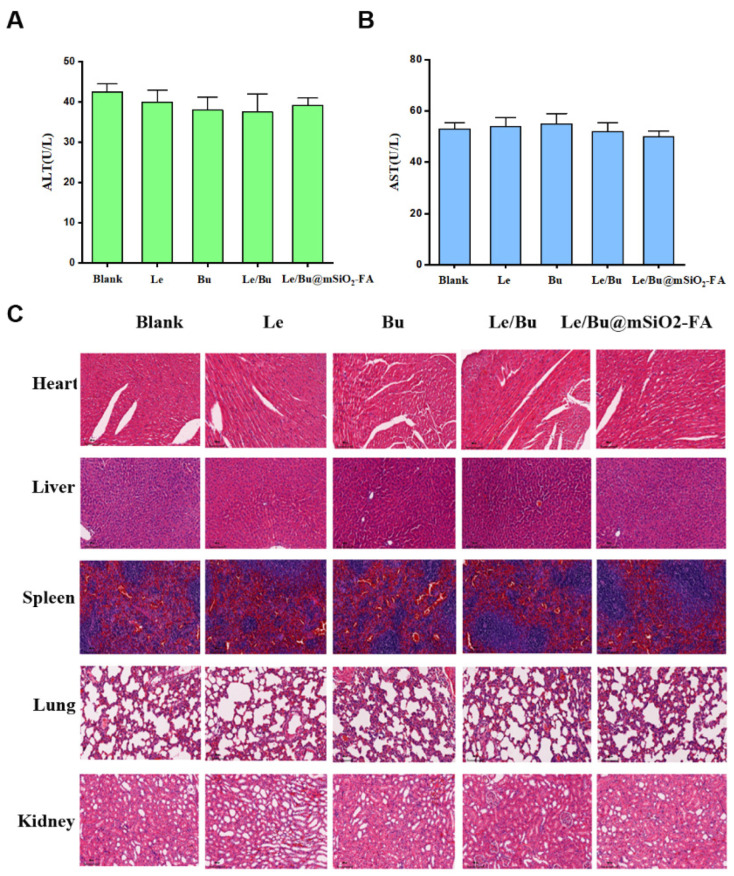
(**A**,**B**) Hematological analysis of the function of liver and heart in heathy mice treated with saline, Le, Bu, Le/Bu, and Le/BumSiO_2_-FA. (**C**) H&E staining of the heart, liver, spleen, lung and kidney after treatment with saline, Le, Bu, Le/Bu, and Le/BumSiO_2_-FA. N = 3 of each group.

Studies have shown that nanoparticles with small particle size can be distributed in the brain, liver and other organs through the blood brain barrier and other biofilms, showing certain toxic effects [45]. In order to evaluate the biosafety of mSiO_2_-FA, the serum alanine aminotransferase (ALT) and aspartate aminotransferase (AST) from healthy mice treated with saline, Le, Bu, Le/Bu, and Le/BumSiO_2_-FA were determined by hematological analysis. The results showed that there was no obvious distinction in ALT and AST content among all groups (Figure 7A,B). Further, the toxic effects of mSiO_2_-FA on the main organs of mice were determined by H&E staining. The results showed that 3 days after mSiO_2_-FA was injected into mice, no obvious pathological changes such as inflammation were observed in the sections of the heart, liver, spleen, lung, kidney, etc. (Figure 7C). All the above results indicate that mSiO_2_-FA has good biological safety.

## 4. Conclusions

In conclusion, a new type of nanoparticle, mSiO_2_-FA, with high-loading Bufalin and Lenvatinib and targeted delivery to tumor tissue was synthesized by a simple method. Bufalin could be loaded into the nanoparticle with high DLE (89.3%) and DLC (17.4%), as could Lenvatinib, with DLE 90.1% and DLC 16.4%. Both drugs release slowly in addition to the high release level (within 72 h, 74.27% and 65.41%, separately). The mSiO_2_-FA nanoparticles loading Bufalin and Lenvatinib could achieve the combination chemotherapy with the synergistic effect from both drugs, the content of which could be modulated separately. Bufalin and Lenvatinib were released into 9810 cells with high efficiency and indicated effective antitumor activity in vitro. Moreover, we observed obvious suppression tumor growth and decreased side effects of drug-related multi-organ toxicity in vivo, because they had the benefits of extended blood circulation, advantageous tumor accumulation, and induced drug release. Consequently, this delivery system of nanoparticle can be expanded to wide combination therapy then be expected to be a potent tool for treating drug-resistant tumors.

## Figures and Tables

**Table 1:** Textural parameters of mSiO_2_. Data are presented as the mean ± standard deviation (SD; *n* = 3).

Sample	Size	BET (m^2^/g)	Dav.Pore (nm)	Vpore (cm^3^/g)
mSiO_2_	112.5 ± 3.9	419.3 ± 1.4	5.8 ± 0.1	0.6 ± 0.1

**Table 2:** Drugs loading data and characterization of the drug loaded mSiO_2_-FA nanoparticles.

Code	Lenvatinib (μg/mL)	Bufalin (μg/mL)	Lenvatinib		Bufalin		D_DLS_ (nm)
			DLE	DLC	DLE	DLC	
1	0	0	/	/	/	/	115
2	100	0	95.3%	16.5%	/	/	121
3	500	0	91.6%	40.3%	/	/	108
4	100	500	93.7%	15.2%	80.3%	39.2%	110
5	100	250	90.1%	16.4%	89.3%	17.4%	113

**Table 3:** IC50 values of different various drug formulations in 9810 cells measured by CCK8 assay.

	Free Lenvatinib	Lenvatinib@mSiO_2_-FA	Bufalin@mSiO_2_-FA	Lenvatinib/Bufalin Free Drugs	Lenvatinib/Bufalin@mSiO_2_-FA
IC50 (μg/mL)	43.26	21.52	22.17	32.14	7.62
SD ^a^	±1.89	±1.26	±2.03	±1.49	±1.06

^a^ Data represent the mean of three separate experiments.

## Data Availability

The data presented in this study are available in the article.
